# The Bioaccessibility of Yak Bone Collagen Hydrolysates: Focus on Analyzing the Variation Regular of Peptides and Free Amino Acids

**DOI:** 10.3390/foods12051003

**Published:** 2023-02-27

**Authors:** Zitao Guo, Yuliang Yang, Bo Hu, Lingyu Zhu, Chunyu Liu, Moying Li, Zhenghua Gu, Yu Xin, Zhongpeng Guo, Haiyan Sun, Yanming Guan, Liang Zhang

**Affiliations:** 1 National Engineering Research Center of Cereal Fermentation and Food Biomanufacturing, Jiangnan University, Wuxi 214122, China; 2 Jiangsu Provincial Research Center for Bioactive Product Processing Technology, Jiangnan University, Wuxi 214122, China; 3 National Engineering Research Center for Functional Food, Jiangnan University, Wuxi 214122, China; 4 Hainan Key Laboratory of Tropical Microbe Resources, Institute of Tropical Bioscience and Biotechnology, Chinese Academy of Tropical Agricultural Sciences, Haikou 571101, China; 5 China National Research Institute of Food and Fermentation Industries Co., Ltd., Beijing 100015, China

**Keywords:** yak bone collagen hydrolysates, bioaccessibility, simulated gastrointestinal digestion and absorption, bioactive peptide, free amino acids

## Abstract

The lack of a bioaccessibility test for yak bone collagen hydrolysates (YBCH) limits their development as functional foods. In this study, simulated gastrointestinal digestion (SD) and absorption (SA) models were utilized to evaluate the bioaccessibility of YBCH for the first time. The variation in peptides and free amino acids was primarily characterized. There was no significant alteration in the concentration of peptides during the SD. The transport rate of peptides through the Caco-2 cell monolayers was 22.14 ± 1.58%. Finally, a total of 440 peptides were identified, more than 75% of them with lengths ranging from 7 to 15. The peptide identification indicated that about 77% of the peptides in the beginning sample still existed after the SD, and about 76% of the peptides in the digested YBCH could be observed after the SA. These results suggested that most peptides in the YBCH resist gastrointestinal digestion and absorption. After the in silico prediction, seven typical bioavailable bioactive peptides were screened out and they exhibited multi-type bioactivities in vitro. This is the first study to characterize the changes in peptides and amino acids in the YBCH during gastrointestinal digestion and absorption, and provides a foundation for analyzing the mechanism of YBCH’s bioactivities.

## 1. Introduction

Collagen peptide is mainly produced by extraction, hydrolysis, and refining with fresh animal tissues which are rich in collagen such as skins, bones, tendons, and scales [1]. In addition to providing a nutritional function, collagen peptide possesses the ability to regulate physiological activities such as modulating immunity, reducing obesity, alleviating osteoporosis, improving bone density, promoting skin health, etc. [2,3,4,5,6]. With people’s the increasing attention to body health, especially since the COVID-19 outbreak, the demand for collagen peptide is increasing. It has been reported that the global collagen peptide market reached USD 598.1 million in 2020 alone. The annual projected growth rate is 5.8% from 2021 to 2028 (https://www.zionmarketresearch.com/report/collagen-peptides-market, accessed on 14 January 2023). Currently, considering the product cost and the fact that peptides are the main components of protein hydrolysates, most collagen peptide in the market is sold in the form of collagen hydrolysates.

Yak bone collagen hydrolysates (YBCH) are manufactured by the enzymatic hydrolysis of yak bone collagen. They are a mixture of peptides and free amino acids. Among these, peptides account for about 88% (*w*/*w*) [7]. YBCH have been widely applied in the fields of food and cosmetics due to its various physiological activities. In recent years, the studies on YBCH have mainly focused on two aspects. One is to obtain bioactive peptides (BAP) through multistage chromatography purification and separation. For example, Ye et al. isolated and identified two novel peptides (GPSGPAGKDGRIGQPG and GDRGETGPAGPAGPIGPV) with osteoblast proliferation-promoting activity by employing an ultrafiltration membrane system and high-performance liquid chromatography-tandem mass spectrometry (HPLC-MS/MS) [8]. In a similar way, it has been reported that antioxidant peptides could be isolated from YBCH [9]. In addition, many researchers and consumers are more interested in the bioactivity of YBCH rather than isolating and purifying BAP from YBCH. Therefore, the other research aim regarding YBCH is to verify its bioactivities through in vivo animal experiments. For example, the immunomodulatory effects of YBCH were tested on cyclophosphamide-induced immunosuppression BALB/c mice [6]. After the intervention, signs related to the immunity of mice indicated that the produced YBCH could effectively prevent and ameliorate immunosuppression by improving innate and adaptive immunity. Additionally, to further certificate the positive effects of YBCH on osteoporosis in vivo, the YBCH were supplemented to ovariectomy-induced osteoporotic rats [5]. After ovariectomy, the osteoporosis-related indices of the rats who received YBCH were significantly ameliorated when compared with those of the control group. The serum untargeted metabolomics revealed that YBCH intake could protect or recover ovariectomy-induced osteoporosis by regulating the amino acid metabolism and the lipid metabolism. In our previous study, the modulating effects of YBCH on the gut microbiota of mice were investigated [10]. After a 30-day intervention, the ratio of Firmicutes to Bacteroidetes in the fecal microbiota was reduced and the amount of short-chain fatty acids in the fecal matter of mice were remarkably elevated. Moreover, the anti-obesity effects of YBCH on high-fat-diet mice were investigated [11]. The joint analysis of the microbiome and untargeted metabolomics suggested that the alleviation effects of YBCH on obesity might be achieved by modulating gut microbiota amino acid metabolism.

In summary, these studies showed that YBCH possess multi-type bioactivities and can be utilized as functional foods or dietary supplements. However, different from polysaccharides, which could resist gastrointestinal digestion and absorption, peptides face severe challenges in the digestive tract. In addition to the harsh gastrointestinal digestive environment, a variety of proteases or peptidases in the digestive fluid further promote the decomposition of peptides [12]. More importantly, the instability of peptides significantly influences their function and metabolism due to the loss of key amino acids or the alteration in peptide size [1]. Therefore, whether for developing functional foods or clarifying the mechanism of their biological effects, it is necessary to evaluate the bioaccessibility of YBCH during gastrointestinal digestion and absorption. Nevertheless, there were fewer reports investigating the changes in YBCH during gastrointestinal digestion and absorption. Owing to their simplicity and universality, simulated gastrointestinal digestion and absorption have been widely implicated in the field of food to predict outcomes of in vivo digestion [13]. Moreover, with the rapid development of peptide identification technology and bioinformatics, a variety of peptide databases and in silico prediction technologies have been developed. These tools make it easier to identify bioactive peptides and analyze their bioactivities [14]. In this study, the objective was to forecast the variation regular of free amino acids and peptides of YBCH during in vivo digestion by employing simulated gastrointestinal digestion and absorption. Moreover, the peptides in the samples were identified by HPLC-MS/MS. The biological activities of the bioavailable peptides were predicted by an in silico analysis and verified by an in vitro test. This study not only provides a foundation for analyzing the mechanism of YBCH’s biological activity but also promotes the application of YBCH in the fields of food and medicine. 

## 2. Materials and Methods

### 2.1. Chemical Agents and Preparation of YBCH

The preparation of YBCH was conducted as previously described [10], and the details as shown in the Supplementary Materials. The pepsin from porcine gastric mucosa (P6887) and pancreatin from porcine pancreas (P7545) were purchased from Sigma-Aldrich Co. (St. Louis, MO, USA). Caco-2 cells and murine macrophage RAW 264.7 cells were bought from the Cell Bank of Chinese Academy of Sciences (Shanghai, China). Angiotensin-converting enzyme (ACE), enzyme from rabbit lung, and dipeptidyl peptidase IV (DPP-IV)-inhibitor screening kit (MAK203) were purchased from Sigma Chemical Co. (St. Louis, MO, USA); Hippuryl-Histidine-Leucine (HHL) and Hippuric Acid (HA) were from Shanghai Maclean Biochemical Technology Co. (Shanghai, China). Cell counting kit (CCK-8) was bought from Dojindo Laboratories (Kumamoto, Japan). The IL-1β assay kit, IL-6 assay kit, TNF-α assay kit, and NO assay kit were purchased from Nanjing Jiancheng Bioengineering Institute (Nanjing, China). Other cell culture-related agents such as Dulbecco’s Modified Eagle Medium (DMEM), 0.25% (*w*/*v*) trypsin-0.91 mM EDTA, and Hank’s balanced salt solution (HBSS) were obtained from Gibco Life Technologies (Grand Island, NY, USA). 1 nmol/µL essential amino acids standard solutions were purchased from Sigma-Aldrich Co. (St. Louis, MO, USA). Unless stated, all other chemicals were analytical-grade and purchased from China Pharmaceutical Group Chemical Reagent Co., Ltd. (Shanghai, China).

### 2.2. Simulated Gastrointestinal Digestion (SD)

The SD was operated as previously described with a slight modification [15]. Briefly, 10 g YBCH was added in 385 mL deionized water. The solution was stirred well and adjusted to pH 2.0 by 1 M HCl. Pepsin was added to the solution in 1:50 (enzyme to the substrate (E/S), *w*/*w*), and the mixture was incubated for 2 h in a water bath shaker (37 °C, 0.01 g) to simulate the gastric digestion. The pepsin activity was terminated by adjusting the pH to 7.0 with 1.0 M NaOH. Then, pancreatin was added into the mixture at the ratio of E/S 1:25. Subsequently, the mixture was placed in the water bath shaker for 2 h (37 °C, 0.01 g) to simulate intestinal digestion. Samples were taken every 30 minutes during the whole process. The simulated digestion was inactivated by incubating the mixture in boiling water for 10 min. After cooling to room temperature and centrifugation (20,000× *g*, 20 min), the supernatant of samples was then lyophilized and stored at −80 °C until further analysis (for a maximum of 2 weeks). 

### 2.3. Simulated Intestine Absorption (SA)

#### 2.3.1. Cell Culture

Caco-2 cells were cultured in a DMEM medium (containing 10% fetal bovine serum, 1% penicillin-streptomycin), and then incubated at 37 °C with 5% CO_2_. The medium was changed every day. The cells were digested and sub-cultured with 0.25% trypsin solution when the cells reached 80–90% confluence. Cells in the logarithmic growth phase were taken for the experiment. Caco-2 cells used in this experiment were 25~35 generations.

#### 2.3.2. Cytotoxicity Test 

The CCK-8 kit was employed to detect the survival rate of Caco-2 cells after obtaining different concentrations of simulated digested YBCH. Caco-2 cells were seeded in a 96-well microplate with a density of 1 × 10^5^ cells/mL. After incubating for 24 h, the culture medium was removed. The Caco-2 cells were randomly divided into a blank control group (only DMEM medium) and the test groups (simulated digested YBCH concentrations were 30 mg/mL, 10 mg/mL, 3.3 mg/mL, 1.1 mg/mL, 0.37 mg/mL, and 0.12 mg/mL). The blank wells only included the same amount of phosphate buffer without cells. Each test was performed in triplicate. After incubation for 6 h, we aspirated the culture medium and added 100 μL of fresh culture medium. Then, 10 μL of CCK-8 was added to each well and incubated for 4 h under 37 °C with 5% CO_2_. After that, the optical density (OD) value of each well was measured by a microplate reader at the wavelength of 450 nm. The cell survival rate was calculated according to the below Formula (1):

(1) $$\mathrm{Cell}\;\mathrm{survival}\;\mathrm{rate}\;\left(\%\right)=\frac{{\mathrm{OD}}_{1}-{\mathrm{OD}}_{3}}{{\mathrm{OD}}_{2}-{\mathrm{OD}}_{3}}\times 100$$

where OD_1_, OD_2_, and OD_3_ are the absorbance of the test groups, blank control group, and blank wells group, respectively.

#### 2.3.3. Transport Studies

The cellular transport study of the simulated gastrointestinal digested YBCH was conducted as previously described with a slight modification [16]. Briefly, the Caco-2 cells were seeded in 24-well transwell inserts (6.5 mm diameter, 0.4 µm pore size, Corning, City, NY, USA) with a density of 1 × 10^5^ cells/cm^2^. The volume of medium on the apical side (AP) and basolateral side (BL) was separately 0.4 mL and 0.6 mL. During the incubation, the medium was refreshed every two days in the first week and every day in the subsequent culture until the monolayer integrity evaluation. The monolayer integrity was evaluated by measuring the transepithelial electrical resistance (TEER) value, detecting paracellular permeability of the fluorescein sodium, and determining the alkaline phosphatase (ALP) activity of the cell culture medium on the two sides of the transwell. The method was operated as previously described [17,18,19]. On the test day, the culture medium on both sides was aspirated and the cell monolayers were rinsed twice with the HBSS. After that, HBSS was added to both sides (AP, 0.4 mL; BL, 0.6 mL) and incubated for 30 min to stabilize prior to the transport studies. Samples (0.4 mL) with a nontoxic concentration (3.3 mg/mL) were added to the AP side, and the fresh blank HBSS solution (0.6 mL) was added to the BL side. After incubation for 2 h, samples on two sides were aspirated and stored at −80 °C until further analysis (for a maximum of 2 weeks). Each test was performed in triplicate.

After the SA, the concentration of peptides on the two sides of the transwell were analyzed by the OPA (o-phthalaldehyde) assay as previously described [20]. The transport rate was calculated using the below Formula (2):

(2) $$\mathrm{transport}\;\mathrm{rate}\;\left(\%\right)=\frac{C_{\mathrm{BL}}\times 0.6\;}{C_{\mathrm{AP}}\times 0.4+C_{\mathrm{BL}}\times 0.6}\times 100$$

where the C_BL_ and C_AP_ are the peptide concentration (mg/mL) on the AP and BL sides after the SA, respectively.

### 2.4. Characterization of the Samples

To comprehensively understand the changes in YBCH during the SD and the SA, the YBCH before and after SD and the solutions on the AP and BL sides at the end of the cell test were harvested and separately termed initial samples (STA), samples after gastrointestinal digestion (SGID), samples on the AP side (SIA), and samples on the BL side (SIB). Then, all these samples were characterized by molecular weight distribution, peptide concentration, free amino acid concentration, and peptides sequence identification. The detecting was conducted as previously described with a slight modification [6,20,21], and the details are provided in the Supplementary Materials. 

### 2.5. Characterization of Bioavailable Bioactive Peptides

#### 2.5.1. In Silico Prediction

The peptides that existed simultaneously in the STA, SGID, and SIA were defined as bioavailable peptides, and their biological activity was predicted by PepRanker (http://distilldeep.ucd.ie/PeptideRanker/, accessed on 14 January 2023) at first [22]. To reduce the number of false positives, the peptides with scores above 0.8 were further calculated and their physicochemical properties (including toxicity, allergenicity, hydrophilicity, hydrophobicity, charge, isoelectric point (pI), and molecular weight) were predicted. Moreover, the nontoxic and non-allergenic peptides were screened to predict their potential ability to be antihypertension peptides (AHTP) [23], antidiabetic peptides (ADP) [24], anti-inflammatory peptides (AIP) [25], and antioxidation peptides (AOP) [26]. The predicting websites are shown in the Supplementary Materials. The novelty of the screened peptides was checked using PepBank (Peptide Database-Search (harvard.edu), accessed on 14 January 2023) and BIOPEP-UWM (http://www.uwm.edu.pl/biochemia/index.php/pl/biopep, accessed on 14 January 2023).

#### 2.5.2. In Vitro Verification

The screened peptides were synthesized by Fmoc solid-phase chemical synthesis (Shanghai RoyoBiotech Co., Shanghai, China). Their structures were verified by LC-MS and the peptide with a purity above 98% was utilized for subsequently verifying their antihypertension, antidiabetic, anti-inflammatory, and antioxidation abilities. The detecting methods were performed as previously described and the details are provided in the Supplementary Materials [27,28,29].

### 2.6. Statistical Analysis

The software SPSS 25.0 (SPSS Inc., Chicago, IL, USA) was employed to analyze the data. One-way ANOVA followed by an LSD test (equal variances assumed) or a Games–Howell test (equal variances not assumed) were performed to evaluate the significant differences between samples. All tests had three replicates. Unless stated, each test was performed in triplicate and the results are presented by mean ± SD. A significant difference was accepted at *p* < 0.05, and *p* < 0.01 was a highly significant difference.

## 3. Results

### 3.1. Molecular Weight Distribution and Concentration of Peptides

**Figure 1:**
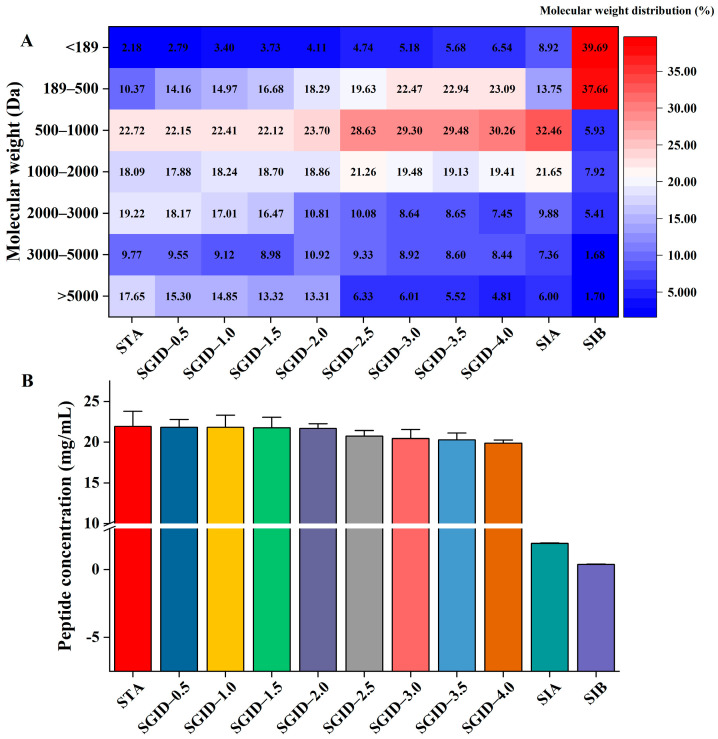
The alteration in the molecular weight distribution (**A**) and concentration (**B**) of peptides during the simulated gastrointestinal digestion and absorption. STA: start digestion samples; SGID-X: samples during simulated digestion and absorption, X is the time (h); SIA: samples on the apical side of the transwell cell; SIB: samples on the basolateral side of transwell cell.

The molecular weight distribution during the SD and the SA is shown in Figure 1A. In the SD, the molecular weight distribution of peptides with a molecular weight below 1000 Da gradually increased, while that of the peptides whose molecular weight was above 5000 Da and 2000–3000 Da decreased. Interestingly, the molecular weight distribution of 3000–5000 Da (around 9%) and 1000–2000 Da (around 19%) was not significantly altered. After the 2 h SA, the molecular weight distribution on the AP side was similar to that at the beginning (SGID-4.0, Figure 1A). Only the molecular weight distribution of 189–500 Da was significantly reduced, from 23.09 ± 2.06% to 13.75 ± 1.09%. For the BL side, the molecular weight distribution of peptides with a molecular weight below 500 Da occupied more than 75%, which indicated that the tripeptides, dipeptides, and free amino acids were the main components of the BL side.

The alteration in peptide concentration was also monitored during the SD and the SA (Figure 1B). The concentration of peptides in the STA was 21.95 ± 1.73 mg/mL, while it was 19.87 ± 0.40 mg/mL after the SD. Although there was a reduction in the concentration of peptides during the SD, statistical analysis indicated that there were no significant differences (*p* > 0.05, Games–Howell test). After the SA, the peptide concentration on the AP and BL side was 1.92 ± 0.04 mg/mL and 0.37 ± 0.04 mg/mL, respectively.

### 3.2. Free Amino Acids Alteration

**Figure 2:**
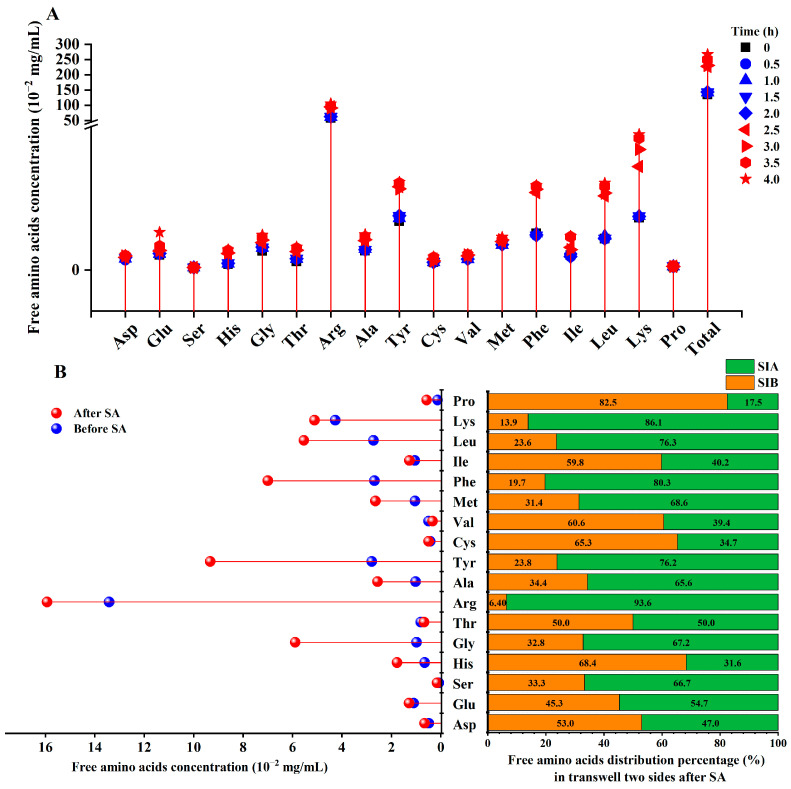
The alteration in free amino acids during simulated gastrointestinal digestion (**A**) and absorption (**B**). Asp: aspartate; Thr: threonine; Ser: serine; Glu: glutamate; Gly: glycine; Ala: alanine; Val: valine; Cys: cysteine; Met: methionine; Ile: isoleucine; Leu: leucine; Tyr: tyrosine; Phe: phenylalanine; Lys: lysine; His: histidine; Arg: arginine; Pro: proline. SIA: samples on the apical side of the transwell cell; SIB: samples on the basolateral side of transwell cell; SA: simulated absorption.

The total concentration of free amino acids increased nearly one time after the SD (from 135 ± 3 × 10^−2^ mg/mL to 266 ± 8 × 10^−2^ mg/mL, Figure 2A). This might be induced by the increase in arginine, tyrosine, phenylalanine, leucine, and lysine (Figure 2A). These kinds of amino acids were the main components of the free amino acids after the SD; the total mass of these free amino acids was above 2.0 mg/mL and their increase ratios were all above 80%. Interestingly, all five kinds of amino acids were not significantly altered during the simulated gastric digestion. However, there was a remarkable increase in the previous 30 min of the simulated intestinal digestion. After that, only a slight increase was exhibited. This phenomenon could be also observed in the alteration in other kinds of amino acids during the SD.

Through comparing the alteration in the concentration of free amino acids before and after SA, it was found that the most increased amino acid was glycine (Figure 2B). The concentration of glycine increased from 0.98 × 10^−2^ mg/mL to 5.89 × 10^−2^ mg/mL; the increase ratio was 501.02%. This was followed by proline and tyrosine, whose increase ratio was 338.46% and 234.41%, respectively. Moreover, leucine, phenylalanine, methionine, alanine, and histidine all increased by more than 100%. The free amino acid distribution on the two sides of the transwell is shown in Figure 2B. It indicates that 82.5% of the proline was transported to the BL side. Noteworthily, only 6.40% of the arginine was distributed on the BL side. These results suggest that intestinal cells might have different transport capacities for different free amino acids in YBCH.

### 3.3. Transport Study 

**Figure 3:**
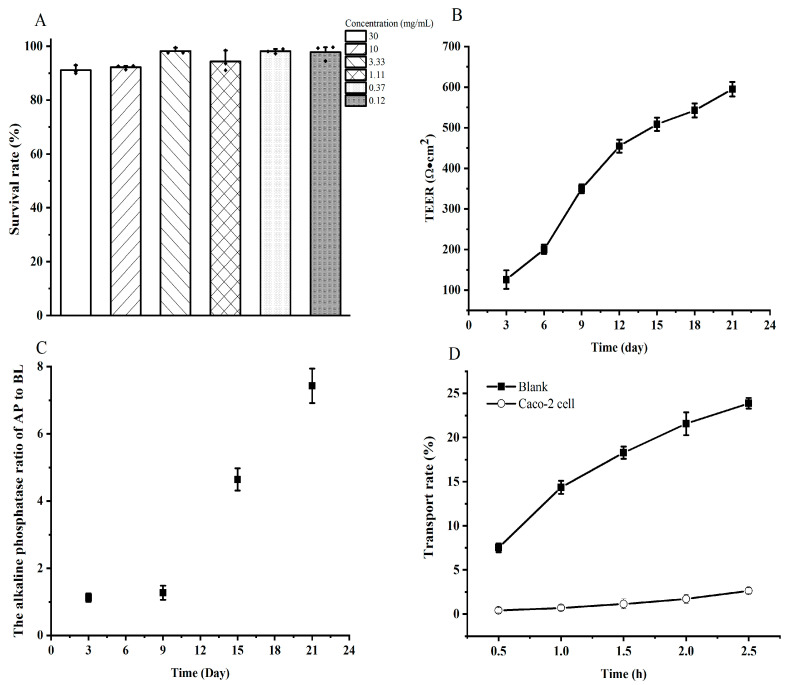
The cell toxicity and cell integrity assessment before transport study. The cell toxicity of different concentrations of digested yak bone collagen hydrolysates (**A**); The transepithelial electrical resistance (TEER) value of Caco-2 cell monolayers (**B**); The alkaline phosphatase (ALP) activity of cell culture medium on the two sides of the transwell (**C**); The permeability of fluorescein sodium on Caco-2 cell monolayers (**D**).

The cell toxicity and monolayer integrity were first studied before the SA. Results indicated that the digested YBCH had no toxicity to the Caco-2 cell. The survival rate of the cell under different concentrations of the digested YBCH was above 90%. Notably, the cell survival rate was almost 100% under the concentration of <3.33 mg/mL (Figure 3A). To avoid inaccurate results of transport and absorption due to the saturation of the peptide during transport, the concentration was set at 3.33 mg/mL for the subsequent SA test. After 21 days of incubation, the TEER value was 595 ± 18.06 Ω·cm^2^ (Figure 3B) and the ALP ratio of the AP to BL was 7.43 ± 0.51 (Figure 3C). Moreover, the paracellular permeability rate of fluorescein sodium was 2.63 ± 0.34%, which was significantly lower than that of the blank control (23.89 ± 0.6%, Figure 3D). These results indicate that the cell monolayer was suitable for the transport study. Calculated according to the concentration of peptides on the two sides (Figure 1B), the transport rate was 22.14 ± 1.58%.

### 3.4. Identification of Peptides

**Figure 4:**
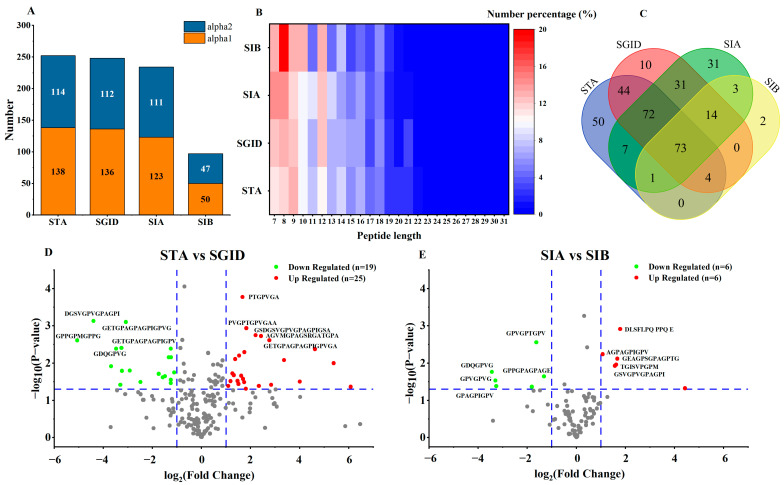
The identification of peptides. The number of peptides in each sample (**A**); The different percentages of peptides with different peptide lengths in each sample, $Number\;percentage\;\left(\%\right)=\frac{Number\;of\;peptides\;with\;X\;peptide\;length}{Total\;number\;of\;peptides\;in\;sample}\times 100\%$, where *X* is the peptide length of the peptides (**B**); The uniqueness and repeatability of peptides in each sample are displayed by the Venn diagram (**C**); The upregulated or downregulated peptides when comparing STA with SGID (**D**) and comparing SIA with SIB (**E**). STA: start digestion samples; SGID-X: samples during simulated digestion and absorption, X is the time (h); SIA: samples on the apical side of the transwell cell; SIB: samples on the basolateral side of transwell cell.

Amongst all samples taken, a total of 440 peptides were identified (information of 440 peptides was uploaded to Mendeley Data, https://data.mendeley.com/datasets/s3j9vpfdff/1, accessed on 23 February 2023, file name: S1-peptideSummary). The number of peptides in STA, SGID, SIA, and SIB was 251, 248, 232, and 97, respectively (Figure 4A). Among these identified peptides, one peptide (PGPAGPAGP) in the STA and two peptides (PGPAGPA and PGAVGPA) in the SIA both belonged to the collagen alpha-1(I) chain and collagen alpha-2(I) chain. The minimized peptide length was 7, while the longest one was 31 (Figure 4B). Among these peptides, the percentage of peptides with lengths ranging from 7 to 15 was more than 75%. The uniqueness and repeatability of peptides in each sample were displayed by the Venn diagram (Figure 4C). The number of peptides that could be identified in both STA and SGID was 193. During the SD, 55 new peptides were produced. Compared with the number of peptides in the SIA, only six new peptides appeared in the SIB after the SA. Significantly, 145 peptides simultaneously existed in the STA, SGID, and SIA. A volcano plot was employed to display the alteration in the relative content of peptides during the SD and SA. Compared with SGID, the relative content of 25 peptides in the STA was significantly upregulated, and that of 19 peptides was downregulated (Figure 4D). In addition, the relative content of 12 peptides was altered in the SIA when compared with that of SIB. Among these peptides, the upregulated and the downregulated contents account for half, respectively (Figure 4E).

### 3.5. Prediction of the Biological Activity of Bioavailable Peptides

BAP can exert their physiological activity in vivo only after they resist decomposition in the digestive tract. Therefore, the 145 peptides that simultaneously existed in the STA, SGID, and SIA deserve more attention. Moreover, 73 of these peptides were also identified in the SIB. These results suggest that the 73 peptides might be absorbed by the intestinal epithelial cells and the other 72 peptides might resist absorption. For more convenient analysis and statistics, the 73 peptides and the 72 peptides were divided into two groups and separately named anti-digestion group (AD) and anti-digestion and anti-absorption group (ADA) (the information of 145 peptides was uploaded to Mendeley Data, https://data.mendeley.com/datasets/s3j9vpfdff/1, accessed on 23 February 2023, file name: S2-basic information of AD and ADA). The bioactive activity of these peptides was predicted by employing in silico tools.

The peptides with a predicted score of biological activity above 0.8 were selected and reordered according to their intensity in the SIA (BAP score of 145 peptides was uploaded to Mendeley Data, https://data.mendeley.com/datasets/s3j9vpfdff/1, accessed on 23 February 2023, file name: S3-properties of AD and ADA). Finally, a total of 6 and 10 typical anti-digestion peptides were screened from the AD group and the ADA group, respectively (Table 1). The shortest peptide length was 7, while the longest one was 19. The molecular weight of these peptides ranged from 571.75 to 1484.94, and most of them were hydrophobic. Moreover, only five peptides were originated from the collagen alpha-2(I) chain. Furthermore, the potential bioactive activity of the nontoxic and non-allergenic peptides was predicted (Table 2). It suggested that all these peptides (except FGFDGDF) might be applied as AHTP, ADP, and AIP. In addition, the antioxidant activity of peptides in the AD and ADA group might be similar. The activity prediction scores of these peptides were very close.

### 3.6. Verification of the Biological Activity of Bioavailable Peptides

To further confirm the prediction results, the biological activities of the screened peptides were verified in vitro. The antihypertension and antidiabetic abilities of these peptides were evaluated by detecting their IC_50_ values on ACE and DPP-IV. For ACE inhibition, only PGPMGPSGPR had an IC_50_ value below 10 mM, while that of other peptides (except FGFDGDF) ranged from 11.7 ± 1.34 mM to 17.07 ± 2.45 mM (Table 3). Considering the application in vivo, such a high IC_50_ value of these peptides suggested their poor ability to develop as ACE inhibitors. The IC_50_ value on ACE of FGFDGDF was not determined due to its low predicted score as AHTP (Table 2). More importantly, we found that FGFDGDF could not be totally dissolved in the reaction solution. Among the seven peptides, GPAGPAGPIGPVG had the best inhibition ability on DPP-IV, with a low IC_50_ value of 0.07 ± 0.01 mM. In addition, GPPGPAGPAG, FGFDGDF, and PAGPAGPIGPV also had a good performance; their IC_50_ values on DPP-IV were lower than 1 mM (Table 3).

**Figure 5:**
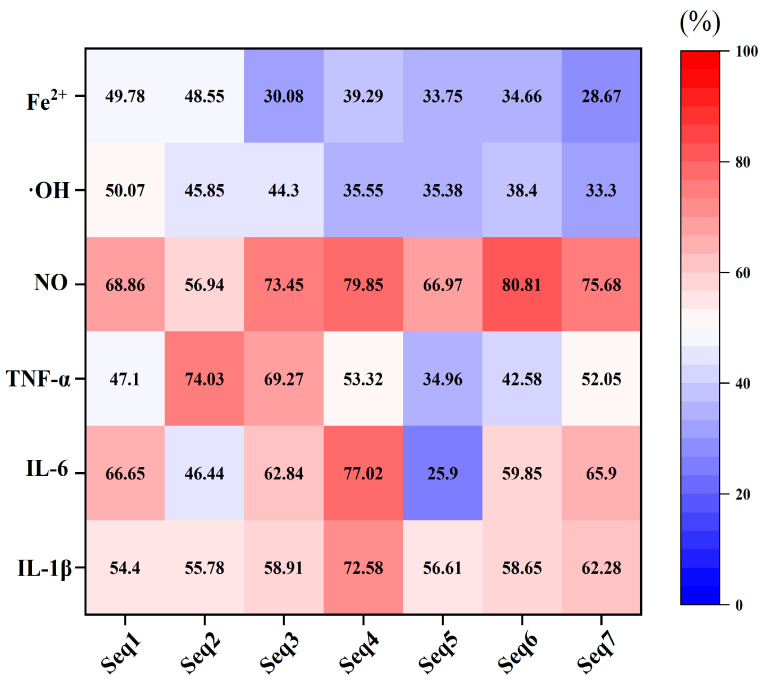
The anti-inflammatory and antioxidant activity in vitro test. Seq1, GGGPGPM; Seq2, GPPGPAGPAG; Seq3, FGFDGDF; Seq4, GPAGPAGPIGPVG; Seq5, AGPAGPAGPAGPR; Seq6, PGPMGPSGPR; Seq7, PAGPAGPIGPV; NO, nitric oxide; TNF-α, tumor necrosis factor-α; IL-6, interleukin 6; IL-1β, interleukin-1 beta; ·OH, hydroxyl radical; Fe2+, ferric ion. The number in each grid presents the mean value of the inhibition rates of inflammatory factors or the antioxidant activity.

To verify the ability of these peptides as AIP, their alleviation effects on the lipopolysaccharide (LPS)-induced murine macrophage inflammation models were evaluated. Results indicated that these peptides had no cytotoxicity on macrophages and they could significantly inhibit the release of inflammatory factors (Figure S1 and Figure 5). The inhibition rates of these peptides on IL-1β and NO were all above 50%. Except for AGPAGPAGPAGPR, which had a low inhibition rate on TNF-α (mean value 34.96%) and IL-6 (mean value 25.9%), most peptides possessed an inhibition rate above 50% on these two inflammatory factors. The hydroxyl radical scavenging activity and ferric ion chelating activity of the seven peptides were detected to characterize their antioxidation ability. Results indicated that the above two activities of the seven peptides were all below 50%. However, consistent with the prediction results (Table 2), there was no difference in the anti-oxidant activity of Seq1 and Seq2 in the AD group, and the analogous situation was also observed between Seq3, Seq4, Seq5, Seq6, and Seq7 in the ADA group (Figure 5).

## 4. Discussion

Bioaccessbility should be paid more attention to when developing new functional products because it is the most important factor affecting the biological activity of functional ingredients. In this study, to further expand the application of YBCH, the bioaccessbility of YBCH was evaluated by employing simulated gastrointestinal digestion and absorption. The changes in peptides and free amino acids were monitored during the SD and the SA. Results indicated that only slight changes in the concentration of peptides and free amino acids were observed (Figure 1 and Figure 2). Moreover, the identification of peptides showed that about 77% of the peptides in the STA could be identified in the SGID after the SD, and about 76% of the peptides in the SGID could be observed in the SIA after the SA (Figure 4C). Although 91 peptides in the SIA were also identified in the SIB, the transport rate of peptides was only 22.14 ± 1.58% (Figure 1B). These results suggest that most peptides in YBCH resist gastrointestinal digestion and absorption. The structural parameters of peptides could significantly influence the bioaccessbility of peptides during digestion and absorption in vivo [12]. From the changes in peptides and free amino acids, we speculated that the stability of YBCH might be attributed to their specific physicochemical properties, including molecular weight and amino acid composition. 

The low molecular weight not only promotes the absorption of peptides but also might be beneficial for the stability of peptides. It has been reported that the low-molecular-weight peptides might avoid protease enzymes due to smaller number of protease recognition and cleavage sites in their sequence [12]. In this study, the peptides with a molecular weight below 3000 Da were the major components of the STA (occupied more than 70%). After the SD, the molecular weight distribution of peptides whose molecular weight was above 3000 Da was finally reduced to around 13.36% (Figure 1A). Thus, a large number of peptides with low molecular weight existing in YBCH might be an important reason for the stability of YBCH during the SD. Moreover, the steric hindrance of these low-molecular-weight peptides might be another important factor to avoid protease or peptidase cleavage. GPAGPPGPIGNV and NAPHMR are BAPs derived from YBCH and sea cucumber gonad, respectively [30,31]. It has been reported that they could resist simulated gastrointestinal digestion. The steric hindrance value of these two BAPs was found to be 0.57 and 0.52 by calculating with an in silico tool named AntiCP 2.0 (https://webs.iiitd.edu.in/raghava/anticp2/predict.php, accessed on 14 January 2023) [32]. However, most of the 145 peptides which were found simultaneously in the STA, SGID, and SIA possessed a higher predicted steric hindrance value than that of the above-reported BAPs (steric hindrance predict score of 145 peptides was uploaded to Mendeley Data, https://data.mendeley.com/datasets/s3j9vpfdff/1, accessed on 23 February 2023, file name: S3-properties of AD and ADA). Therefore, the steric hindrance of these low-molecular-weight peptides might increase the difficulty of the enzymatic hydrolysis process during gastrointestinal digestion. 

In addition, peptides with different molecular weights might have their own characteristics during gastrointestinal digestion and absorption. For example, during simulated gastric digestion, the molecular weight distribution of the peptides with a molecular weight above 5000 Da was only reduced from 17.65 ± 1.23% to 13.31 ± 0.72%. However, at the beginning of the simulated intestinal digestion, the molecular weight distribution of these peptides sharply decreased from 13.31 ± 0.72% to 6.33 ± 0.37%. On the contrary, for the peptides with a molecular weight 2000–3000 Da, the molecular weight distribution was reduced by half (19.22 ± 0.91% to 10.81 ± 1.15%) during the simulated gastric digestion, while only a slight decrease (10.08 ± 0.54% to 7.45 ± 0.49%) was observed in the simulated intestinal digestion (Figure 1). Additionally, the molecular weight could not only influence the permeability rates of peptides but also affect their absorption pathway. Many reports have suggested that the molecular weight of peptides has a negative relationship with the permeability rates [33,34]. This might be attributed to their different path through intestinal endothelial cells. For example, most di- and tripeptides were transported via PepT1, which is a member of the H^+^-dependent carried family and widely distributed on intestinal endothelial cells. The larger ones might be absorbed through a paracellular route via tight junctions or transcytosis [35]. However, it should be noted that the paracellular diffusion area only occupied 0.01% of the human gut surface area, and transcytosis is an energy-dependent transport route [36,37]. Therefore, in fact, most of the peptides could not be effectively absorbed by the intestinal epithelial cells. Recently, it has been difficult to accurately quantify and identify the small peptides from the protein hydrolysates, especially for di- and tripeptides [38]. In this study, limited by the detection technology, no peptides with a length below seven were identified. However, it could be speculated that most di-/tripeptides (molecular weight below 500 Da) in the YBCH were absorbed by the Caco-2 cell monolayers based on the molecular weight distribution of the peptides in the SIB and the SIA (Figure 1A). Meanwhile, the major parts of YBCH, peptides with molecular weight ranging from 500 Da to 2000 Da, were not altered significantly during the SA. 

The number and location of different amino acids in the peptide sequence could significantly alter the bioavailability of the peptide. Especially for collagen-derived peptides, it is necessary to pay more attention to the effects of proline and glycine on the bioaccessibility of peptides. This is mainly because there are a large number of repeated “G-X-Y” structures in collagen alpha chains, and the X and Y usually are occupied by proline or hydroxyproline [1]. Proline and hydroxyproline have long been recognized as important factors to increase the stability of peptides during gastrointestinal digestion [35]. On the one hand, this might be due to the fact that proline and hydroxyproline are not the cleavage sites of digestive proteases and peptidases including pepsin, trypsin, and chymotrypsin, etc. [12]. During the SD and the SA, free proline content was very low (<0.01 mg/mL, Figure 2). Inversely, the amino acids which are the cleavage sites for most proteases, such as arginine, lysine, phenylalanine, leucine, and tyrosine, were the major components of the free amino acids (Figure 2). On the other hand, the proline has a γ-lactam structure. This structure is similar to the γ-lactam moiety in the pyroglutamyl peptides, which could increase the steric hindrance of peptides to proteases [39,40]. In this study, the composition of peptide sequences in STA showed that only 15 peptides did not contain proline. The number of peptides containing two, three, and four prolines in their sequence was 55, 86, and 50, respectively. Notably, there were seven peptides that contained eight prolines in their sequence (Supplementary Materials, Figure S2). Therefore, the high amount of proline in their sequence might enhance the steric hindrance of these peptides to protease and peptidase. In addition, prior studies have noted the effects of different amino acids located in the N- and C-terminal of peptides on their ability to resist digestion and absorption. For example, the peptides with an N-terminal containing isoleucine, lysine, methionine, proline, and valine or a C-terminal containing valine have high permeability [41]. In this study, a total of 125 peptides in STA had glysine in their N-terminal, which accounted for 49.8% of all peptides in STA. In the C-terminal of the peptides in STA, the most widespread amino acid was alanine (57 peptides), followed by arginine (46 peptides) and glycine (45 peptides) (Supplementary Materials, Figure S3). Although there were 26 peptides with C-terminals containing valine, it has been reported that the amino acids in the N-terminal contribute more to bioaccessbility than those in the C-terminal [40]. Therefore, the absence of permeability-promoting amino acids in the N-terminal of peptides in YBCH might enhance the ability of these peptides to resist digestion and absorption.

Moreover, we found that the difference in molecular weight and amino acid composition might explain why the 73 peptides in the AD group might be absorbed by the intestinal epithelial cells and the other 72 peptides in the ADA group might resist absorption. Regarding molecular weight (Supplementary Materials, Figure S4A), the number of peptides with a molecular weight below 1000 Da was 44 in the AD group, while that in the ADA group was 36. Meanwhile, there was a lower number of peptides with molecular weight between 1000 Da and 1500 Da in the AD group. Regarding peptide length (Supplementary Materials, Figure S4B), it was found that the peptide length of most of the peptides in the AD group was located between 7 and 10, which accounts for about 60%. These results indicated that the molecular weight of peptides in the AD group might be lower than that in the ADA group with the same peptide length. Thus, the higher molecular weight in the ADA group than that in the AD group might be one reason for the peptides’ different performance during intestinal epithelial cell absorption. After statistical analysis, there were 15 peptides in the AD group with C-terminal-containing valine, which accounts for about 20.5% of the total number of peptides in the group (Supplementary Materials, Figure S4C). However, that number was five in the ADA group, which accounts for only 6.9%. Thus, the high ratio of peptides with C-terminal-containing valine in the AD group might contribute to their absorption during SA. To sum up, the molecular weight and amino acid composition of the peptides might be two important reasons that influence their bioaccessibility during gastrointestinal digestion and intestinal epithelial cell absorption.

The traditional screening process of BAP usually requires multiple purification steps combined with in vitro activity verification to continuously narrow the search area in the protein hydrolysates. Finally, the sequence composition of the peptides was determined by LC-MS/MS. Moreover, this procedure needs a lot of time, and more important is that the obtained BAP be tested to evaluate its ability to resist digestion if it is developed as a functional ingredient. This screening method might cause the obtained BAP to be unsuitable for in vivo use. By constructing a reliable simulated in vitro digestion and absorption model, the changes in peptides during the entire digestion and absorption process can be effectively monitored. This not only helps us comprehensively know about the characteristics of peptides during digestion but also obtain results that are closer to the real situation in vivo. Then, combined with the current increasingly mature in silico tools, we can predict the desired bioactivity and further conduct in vitro and in vivo verification. This method might be more efficient and reliable. In this study, although limited by the detection technology in that the peptides below hepteptide were not identified, 440 peptides were obtained in all taken samples. This undoubtedly gives us a treasure trove of many peptides which deserve a further explore into their bioactivity. The most interesting finding was the 145 peptides that existed from the start to the end of the simulated digestion and absorption. After the theoretical calculation and in silico prediction, two typical anti-digestion BAPs and five typical anti-digestion and -absorption BAPs were separately screened (Table 1 and Table 2). After in vitro activity verification, some peptides showed that they deserved further testing in vivo. In addition, combined with our previous work, their bioactivity and their effects on the composition of gut microbiota should be further investigated and validated. 

## 5. Conclusions

The bioaccessibility test of functional ingredients is very important for illustrating their mechanisms of action and promotion in the application field. Although YBCH have been developed as dietary nutrients and functional foods for a long time in China due to their various biological activities such as antioxidant and immunomodulation effects, their bioaccessibility has not been reported. Notably, fewer reports exist regarding the variation regular of free amino acids and peptides during gastrointestinal digestion and adsorption, which limits the application of YBCH. Different from previous studies focused on investigating the activity of YBCH, in this study, simulated gastrointestinal digestion and absorption were conducted to characterize the alteration in peptides and free amino acids of YBCH in the digestive tract. Results indicated that most peptides in YBCH resist digestion and absorption, which might be due to the low molecular weight of peptides, the high-frequency distribution of proline, and the absence of permeability-promoting amino acids (high glycine in the N-terminal and less valine in the C-terminal) in the terminal of peptides. Moreover, the joint utilization of in silico analysis and in vitro tests suggested that various types of BAPs could be obtained after the YBCH digestion and absorption. This study promotes new insights into the application of YBCH, and the bioactivities of the obtained peptides should be further verified in the future.

## Figures and Tables

**Table 1:** The physicochemical properties and biological activity of anti-digestion and -absorption peptides.

	Amino Acid Sequence	BAP_Score	Intensity (×109)	Length	Mol wt	Hydrophobicity	Hydrophilicity	Charge	pI	Collagen Type	Toxin	Allergen
AD	GEAGPAGPAGPAGPR	0.81	726.48	15	1261.56	−0.06	0.27	0	6.00	alpha-2	Non	Yes
	GGGPGPM	0.90	233.89	7	571.75	0.11	−0.19	0	5.52	alpha-2	Non	Non
	AGPAGPIGPV	0.80	38.67	10	835.11	0.2	−0.43	0	5.57	alpha-1	Non	Yes
	GPPGPAGPAG	0.88	19.90	10	776.98	0.06	−0.13	0	5.52	alpha-1	Non	Non
	GPPGPMGPPGLAGPPGESG	0.89	19.69	19	1629.1	0.04	−0.02	−1	4.00	alpha-1	Non	Yes
	GPPGPMGPPG	0.95	15.40	10	863.14	0.09	−0.1	0	5.52	alpha-1	Non	Yes
ADA	AGPAGPAGPAGPR	0.88	126.00	13	1075.36	−0.03	0.08	1	9.79	alpha-2	Non	Non
	FGFDGDF	0.94	57.56	7	803.91	0.1	−0.21	−2	3.56	alpha-2	Non	Non
	PAGPAGPIGPV	0.83	20.76	11	932.24	0.18	−0.39	0	5.96	alpha-1	Non	Non
	GPPGPMGPPGLA	0.95	6.78	12	1047.41	0.11	−0.3	0	5.52	alpha-1	Yes	Non
	PGPMGPSGPR	0.87	3.71	10	952.23	−0.16	0.2	1	10.18	alpha-1	Non	Non
	GPAGPAGPIGPVG	0.90	2.54	13	1046.38	0.18	−0.33	0	5.52	alpha-1	Non	Non
	GFDGDFY	0.86	2.49	7	819.91	0.02	−0.19	−2	3.56	alpha-2	Non	Yes
	GPPGPMGPPGLAGPPGE	0.91	1.94	17	1484.94	0.05	−0.04	−1	4.00	alpha-1	Yes	Non
	GPAGPAGPIGPV	0.87	0.92	12	989.31	0.18	−0.36	0	5.52	alpha-1	Non	Yes
	GPPGPMGPPGL	0.97	0.87	11	976.32	0.1	−0.28	0	5.52	alpha-1	Yes	Yes

Note: AD: anti-digestion group; ADA: anti-digestion and anti-absorption group; BAP_score: biological activity predicted score; Intensity: the mean value of the relative content of peptides in the SIA; Toxin: toxin prediction; Allergen: allergen prediction; Mol wt: molecular weight; pI: isoelectric point. Only the BAP_score of peptides above 0.8 are shown.

**Table 2:** The predicted bioactivity of nontoxic and non-allergenic peptides.

	Amino Acid Sequence	AHTP (Score)	ADP (Score)	AIP (Score)	FRS (Score)	CHEL (Score)
AD	GGGPGPM	Yes (0.90)	Yes (0.83)	Yes (0.38)	0.56	0.25
	GPPGPAGPAG	Yes (0.99)	Yes (0.89)	Yes (0.47)	0.50	0.24
ADA	FGFDGDF	Non (0.12)	Yes (0.82)	Yes (0.44)	0.42	0.24
	GPAGPAGPIGPVG	Yes (0.99)	Yes (0.94)	Yes (0.47)	0.46	0.21
	AGPAGPAGPAGPR	Yes (0.99)	Yes (0.59)	Yes (0.40)	0.55	0.22
	PGPMGPSGPR	Yes (0.95)	Yes (0.69)	Yes (0.36)	0.49	0.23
	PAGPAGPIGPV	Yes (0.98)	Yes (0.89)	Yes (0.48)	0.47	0.22

Note: AD: anti-digestion group; ADA: anti-digestion and anti-absorption group; AHTP: antihypertension peptides; ADP: antidiabetic peptides; AIP: anti-inflammatory peptides; FRS: free radical scavenging activity; CHEL: metal ion chelating activity.

**Table 3:** The IC_50_ values on ACE and DPP-IV of the seven screened bioavailable peptides.

IC_50_ (mM)	Seq1	Seq2	Seq3	Seq4	Seq5	Seq6	Seq7
ACE	16.12 ± 2.11 ^a^	13.13 ± 1.63 ^ab^	ND	17.07 ± 2.45 ^a^	11.7 ± 1.34 ^b^	7.73 ± 0.54 ^c^	15.42 ± 2.08 ^a^
DPP-IV	1.26 ± 0.15 ^b^	0.82 ± 0.09 ^c^	0.59 ± 0.07 ^d^	0.07 ± 0.01 ^f^	4.56 ± 0.57 ^a^	3.74 ± 0.52 ^a^	0.24 ± 0.03 ^e^

Note: ACE, angiotensin-converting enzyme; DPP-IV, dipeptidyl peptidase IV; Seq1, GGGPGPM; Seq2, GPPGPAGPAG; Seq3, FGFDGDF; Seq4, GPAGPAGPIGPVG; Seq5, AGPAGPAGPAGPR; Seq6, PGPMGPSGPR; Seq7, PAGPAGPIGPV; ND, not detected. Each assay has three parallels and the results are presented as mean ± SD. Different alphabets indicated there was a significant difference between samples.

## Data Availability

Data are contained within the article.

## Supplementary Materials

The following supporting information can be downloaded at: https://www.mdpi.com/article/10.3390/foods12051003/s1, Figure S1: The screened out seven peptides on the toxicity of murine macrophages; Figure S2: The number of prolines in the peptide sequence; Figure S3 The terminal residue of peptides in start digestion samples; Figure S4 The statistical analysis of molecular weight distribution (A), peptide length (B), and amino acids composition (C) of peptides in the AD group and ADA group.
