# Supplementary material for: The Bioaccessibility of Yak Bone Collagen Hydrolysates: Focus on Analyzing the Variation Regular of Peptides and Free Amino Acids
Source: Foods. 2023 Feb 27;12(5):1003. doi: 10.3390/foods12051003 (PMC10001269; doi:10.3390/foods12051003)
Supplement: Supplementary file 1 [file foods-12-01003-s001.zip › foods-2191215-supplementary.pdf]

The bioaccessibility of yak bone collagen hydrolysates: focus on analyzing the variation regular of peptides and free amino acids

Zitao Guo [1,2], Yuliang Yang [1,2], Bo Hu [3], Lingyu Zhu [1,2], Chunyu Liu [1,2], Moying Li [1,2], Zhenghua Gu [1,2], Yu Xin [1,2], Zhongpeng Guo [1,2], Haiyan Sun [4], Yanming Guan [5], Liang Zhang [1,2,*]

[1.] National Engineering Research Center of Cereal Fermentation and Food Biomanufacturing, Jiangnan University, Wuxi 214122, China

[2.] Jiangsu Provincial Research Center for Bioactive Product Processing Technology, Jiangnan University, Wuxi 214122, China

[3.] National Engineering Research Center for Functional Food, Jiangnan University, Wuxi 214122, China

[4.] Hainan Key Laboratory of Tropical Microbe Resources, Institute of Tropical Bioscience and Biotechnology, Chinese Academy of Tropical Agricultural Sciences, Haikou 571101, China

[5.] China National Research Institute of Food and Fermentation Industries Co., Ltd., Beijing 100015, China

* Corresponding author

E-mail: zhangl@jiangnan.edu.cn;

Materials and methods

1. Preparation of YBCH

Food-grade alcalase, neutrase, and flavourzyme were mixed with ratio 2:2:0.5 and introduced into the collagen powder solution to initiate the hydrolysis. The enzyme/substrate (yak bone collagen powder) ratio was 3: 1000 (w/w). The hydrolysis was conducted for 4 h at 55 ± 0.5 °C and terminated by heating at 95 °C for 20 min. Then, the mixture was centrifugated at 10,000 g for 15 min after cooled to room temperature. The supernatant was collected and concentrated by rotated-vapor. After that, the concentrated products were lyophilized and stored at -20 °C until use (for a maximum of 2 weeks).

2. Characterization of the samples

2.1 Molecular weight distribution and peptide concentration.

The molecular weight distribution of samples was detected as previously described (Gao et al., 2019). Briefly, the size exclusion HPLC (SE-HPLC) systems (Waters, Massachusetts, USA) were employed and the mobile phase buffer was prepared by dissolving acetonitrile (AN, 45%, v/v) and trifluoroacetic acid (TFA, 0.1%, v/v) in ultrapure water. The freeze-dried simulated digested YBCH was dissolved by the TFA-AN buffer to 1 mg/mL. The Caco-2 cell test solutions of the two sides were diluted three times by the TFA-AN buffer and filtered by 0.22 μm syringe filter before the test. A TSK gel G2000 SWXL column (7.8×300 mm, TOSOH, Tokyo, Japan) was employed in the detection. Aliquots of 10 μL samples were eluted by TFA-AN buffer at the flow rate of 0.5 mL/min and the column temperature was 40 °C. The UV detector was used to monitor elution components at 214 nm. Gly-Gly-Gly (189 Da), Gly-Gly-Tyr-Arg (451 Da), bacitracin (1,422 Da), aprotinin (6,511 Da) and Cytochrome C (12,362 Da) were applied as

standards to get a calibration curve of molecular weight. The peptide concentration in each sample was determined as previously described (Puchalska, Concepcion Garcia, & Luisa Marina, 2014).

2.2 Free amino acids concentration.

Agilent 1100 HPLC system (Agilent Technologies Inc., California, USA) and the Agilent Hypersil ODS column (5 µm, 4.0 × 250 mm) were employed to test the free amino acids. The mobile phase buffer A (pH 7.2) was composed of sodium acetate (27.6 mmol/L), triethylamine, and tetrahydrofuran at the volume ratio of 500: 0.11: 2.5, respectively. The mobile phase buffer B (pH 7.2) was formed by sodium acetate (80.9 mmol/L), methanol, and AN with a volume ratio of 1: 2: 2. The linear gradient was designed as follows: 0 min, 8% B; 17 min, 50% B; 20.1 min, 100% B; 24 min, 0% B. The flow rate was 1.0 mL/min and the column temperature was 40 °C. The eluted amino acids were detected under 338 nm by a UV detector (UV1700, Shimadzu Corp., Kyoto, Japan, proline was 262 nm). 1 g of the YBCH before and after SGID was dissolved by 25 mL trichloroacetic acid (TCA, 5% (w/v)). The solution was statically placed 2 h after ultrasonic for 20 min under room temperature. After that, the samples were filtered by a 0.22 µm syringe filter before loading to the column. The solutions in the AP and BL sides of the cell test were diluted by the same volume of 10% (w/v) TCA and the diluted samples were filtered by a 0.22 µm syringe filter before loading to the column. The external standard method was used for quantification.

2.3 Identification of peptides

The peptides identification was operated as previously described with a modification (Ma et al., 2021). At first, the total peptide concentration of the samples was adjusted to below 10 mg/mL by using 8 M urea. The dithiothreitol (DTT) was added to the samples to a final concentration of 10 mM and the samples were incubated at 56 °C for 30 min. Then, iodoacetamide (IAM) was

incorporated with samples to a final concentration of 55 mM and reacted for 45 min at 25 °C in a dark room. After that, the peptides with molecular below 10 kDa in samples were obtained by desalting with a C18 column and filtering with a 10 kDa ultra-centrifugal filter. Then, the obtained peptides were lyophilized. Next, the peptide identification was performed on an LC-20A HPLC system (Shimadzu, Kyoto, Japan) coupled with a mass spectrometry system (Q-Exactive, Thermo Scientific, San Jose, CA). The detailed settings of the HPLC-MS/MS system and raw data processing methods were given in the supplemental materials.

The mobile phase was composed by buffer A (5% AN, 0.1% formic acid (FA)) and buffer B (95% AN, 0.1% FA). The freeze-dried peptides were dissolved by buffer A to 35 µL. Then, 30 µL supernatants were obtained after centrifugation (20,000 *g*, 10 min) and loaded onto a trap column (300 µm × 5 mm, µ-Precolumn, Thermo Scientific) connected to an analytical column (75 µm × 25 cm, 3-µm particle). The linear gradient was set as follows: 5% buffer B for 5 min; 5% - 35% buffer B in 35 min; 35% - 60% buffer B in 5 min; 60% - 80% buffer B in 2 min and held for 2 min; 80% - 5% buffer B in 1 min and held for 10 min. The separated components were transferred to the mass spectrometry system and detected under the data-dependent acquisition (DDA) model. The main parameters were designed as follows: the mass scan was 350-1600 *m*/*z*. The resolution was 70,000 and the automatic gain control (AGC) target was set to $3e^6$. The tandem mass spectrometry scan was 100 *m*/*z*, and the resolution was 17,500 with the AGC target was $1e^5$.

The obtained raw data were analyzed by using MaxQuant (version: 1.5.3.30; https://www.maxquant.org/maxquant/, access date: 2023/01/14). The database was composed of a collagen alpha-1(I) chain (Reference Sequence: XP_005890387.1) and a collagen alpha-2(I) chain (Reference Sequence: XP_005909757.1) that downloaded from NCBI (https://www.ncbi.nlm.nih.gov/, access date: 2023/01/14). The following parameters of MaxQuant

were set: enzyme was trypsin; minimal peptide length was 7; peptide mass tolerance was 4.5 ppm; fragment mass tolerance = 20 ppm; carbamidomethyl for fixed modification; oxidation of methionine and N-terminal for variable modifications. Maximum false discovery rates (FDR) were set to 1% on peptide spectrum match level. MaxQuant was used to extract the peak area and calculate the quantitative value of peptides. After data normalization, the fold change of peptides between the comparison group was calculated. Welch's T-test was used for the significance test. Furthermore, the fold change ≥ 2.0 and p-value < 0.05 were used as the judging criteria for screening the peptides with significant differences between the two groups.

3. Characterization of bioavailable bioactive peptides

3.1 The predicting websites

Anti-hypertension peptides (AHTP): http://thegleelab.org/mAHTPred/; access date: 2023/01/14

Anti-diabetic peptides (ACP): http://pmlabstack.pythonanywhere.com/StackDPPIV; access date: 2023/01/14

Anti-inflammatory peptides (AIP): http://thegleelab.org/AIPpred/index.html; access date: 2023/01/14

Anti-oxidant peptides (AOP): https://services.healthtech.dtu.dk/service.php?AnOxPePred-1.0; access date: 2023/01/14

3.2. *In vitro* activity assay

3.2.1 Anti-hypertension

The ACE inhibitory activity of the synthesized peptides was employed to characterize the anti-hypertension effects of the screened peptides. The detecting was conducted as previously reported with some modifications(Zhao, Zhang, Tao, Chi, & Wang, 2019). First, ACE, HHL and different concentrations of the synthesized peptides were prepared in 0.1 mol/L borate buffer

solution (BBS) (pH 8.3, containing 0.3 mol/L NaCl). Then, 50 μL of sample solution and 100 μL of HHL solution (3 mmol/L) were mixed and preincubated at 37°C for 10 min. Next, 50 μL of ACE (10 mU/mL) were added to start the reaction. After incubation for 40 min at 37 °C, the reaction was terminated by adding 150 μL of HCl (1 M). The HA content of mixture was determined by using a high-performance liquid chromatography (HPLC) system (Agilent 1260, Agilent Technologies, CA, USA) with Zorbax SB C18 column (4.6 mm ×150 mm, 5 μm). The column was eluted by isocratic mobile phase A (0.1% TFA in acetonitrile) and B (0.1% TFA in ultrapure water) at a flow rate of 0.5 mL/min and a constant ratio (A: B= 25: 75). The injected volume was 10 μL, column temperature was 30°C, and the effluent was monitored at 228 nm. The control group used an equal volume of buffer instead of the sample solution. The ACE-inhibitory activity (%) was calculated by using the following equation (1):

$$\text{ACE inhibition rate (\%)} = \frac{(\mathrm{A} - \mathrm{B})}{\mathrm{A}} \times 100\% \qquad (1)$$

Where A was the peak area of HA in the control group, and B was the peak area of HA in the sample group.

The IC50 value was defined as the required concentration of inhibitor producing 50% of inhibition of ACE.

3.2.1 Anti-diabetic

The DPP-IV inhibitory activity of the synthesized peptides was employed to characterize the anti-diabetic effects of the screened peptides. The detecting was performed by using a DPP-IV inhibitor screening assay kit following the manufacturer’s instructions. At first, DPP-IV, substrate, positive control (sitagliptin), and the different concentration of synthetic peptides (0, 0.5, 1, 2, 4, 8 mmol/L) were prepared by dissolving with the buffer solution. Then, peptide samples (25μL) dispersed in assay buffer at different concentrations. Next, 49 μL of mixture and 1μL of DPP-IV

were added in a 96-well plate, and preincubated at 37°C for 10 min in dark. Additionally, mixture without DPP-IV and with sitagliptin were prepared as negative control and positive control, respectively. After that, 23 μL of buffer and 2 μL of DPP-IV substrate solution (Gly-Pro-Aminomethylcoumarin) was added to each well. Reactions were incubated at 37°C for 15 min and inhibition was measured every 1 min for 20 min. The fluorescence value was detected with excitation of 360 nm and emission of 460 nm. The inhibition rate of each sample was calculated as follows:

$$\text{Relative inhibition activity (\%)} = \frac{(\text{Slope}_1 - \text{Slope}_2)}{\text{Slope}_1} \times 100\% \qquad (2)$$

$$\text{Slope} = \frac{\text{FLU}_2 - \text{FLU}_1}{\text{T}_2 - \text{T}_1}$$

where Slope_1 was the slope of the control and Slope_2 was the slope of the sample; FLU was the fluorescence value and T was the detecting time.

3.2.3 Anti-inflammatory

Macrophage cell culture

Murine macrophage RAW 264.7 cell was cultured in a DMEM growth medium that supplemented with 10% FBS and 1% penicillin-streptomycin. RAW 264.7 cells were plated at densities 1×10^6 cells in 75 cm^2 tissue culture plates and cultured overnight in a humidified incubator at 37 °C and 5% CO_2 atmosphere. After two to three days of culture, the cells accounted for more than 80% of the plate area were subcultured. When the cells grew well, they were frozen and stored in liquid nitrogen.

Cell viability assay

Cells were seeded in a 96-well plate at a density of 3×10^4 cells/well and allowed to grow under the above-described conditions to confluence (24 h). The cells were exposed for 2 h in the 100 μL serum-free medium that containing different concentration of peptides (3.7, 11.1, 33.3, 100,

300 nmol/mL). Then, 10μL serum-free medium containing lipopolysaccharide (LPS, 11 μg/mL) was added to the medium and the cells were incubated for 24 h at 37 °C, 5% CO_2. After treatment, cell viability was determined using the CCK-8 cell viability assay kit. Briefly, 10 μL of CCK-8 solution was added to the medium. After 2 h incubation, the absorbance of medium was recorded at 500 nm in a TECAN SPARK microplate reader (Nanjing Cleande Scientific Instrument Co, Nanjing, China).

Inflammatory factors quantification in macrophages culture medium

All samples were assayed for potential anti-inflammatory activity. Murine macrophage RAW 264.7 cells were seeded in 96-well plates at a density of 3×10^5 cells/well and allowed to grow to confluence (24 h) in a humidified incubator at 37 °C and 5% CO_2 atmosphere. The cells in the test group were pre-treated for 2 h in serum-free medium with peptide fractions (1.11 mg/mL) dissolved, then elicited with LPS at 1 μg/mL for an additional 24 h. The cells in the blank control were treated with serum-free medium without any drugs, while the cells in the positive control were only treated with LPS. The supernatant of the medium was collected after centrifugation 5 min at 702 *g*, 4 °C. The collected supernatants were stored in a refrigerator at -80 °C until further analysis.

The concentration of IL-1β、IL-6、TNF-α and NO were determined by using the commercial test kits following the manufacturer's instruction. The inhibition rate of inflammatory factors (IL-1β, IL-6, TNF-α and NO) was calculated by the following equation (3):

$$\text{Inhibition rate (\%)} = \left(1 - \frac{\text{Asample} - \text{Ablank}}{\text{Apositive} - \text{Ablank}}\right) \times 100\% \qquad (3)$$

where A_{blank} is the concentration of inflammatory factors in the blank group, A_{sample} is the concentration of inflammatory factors in the test group, $A_{positive}$ is the concentration of inflammatory factors in the positive group.

3.2.4 Anti-oxidation

The hydroxyl radical scavenging activity and metal ion chelating activity of the synthetic peptides were detected to characterize the ability of anti-oxidation. The detecting was performed as previously described (Venkatesan & Nazeer, 2013).

Results

1. The cell toxicity test

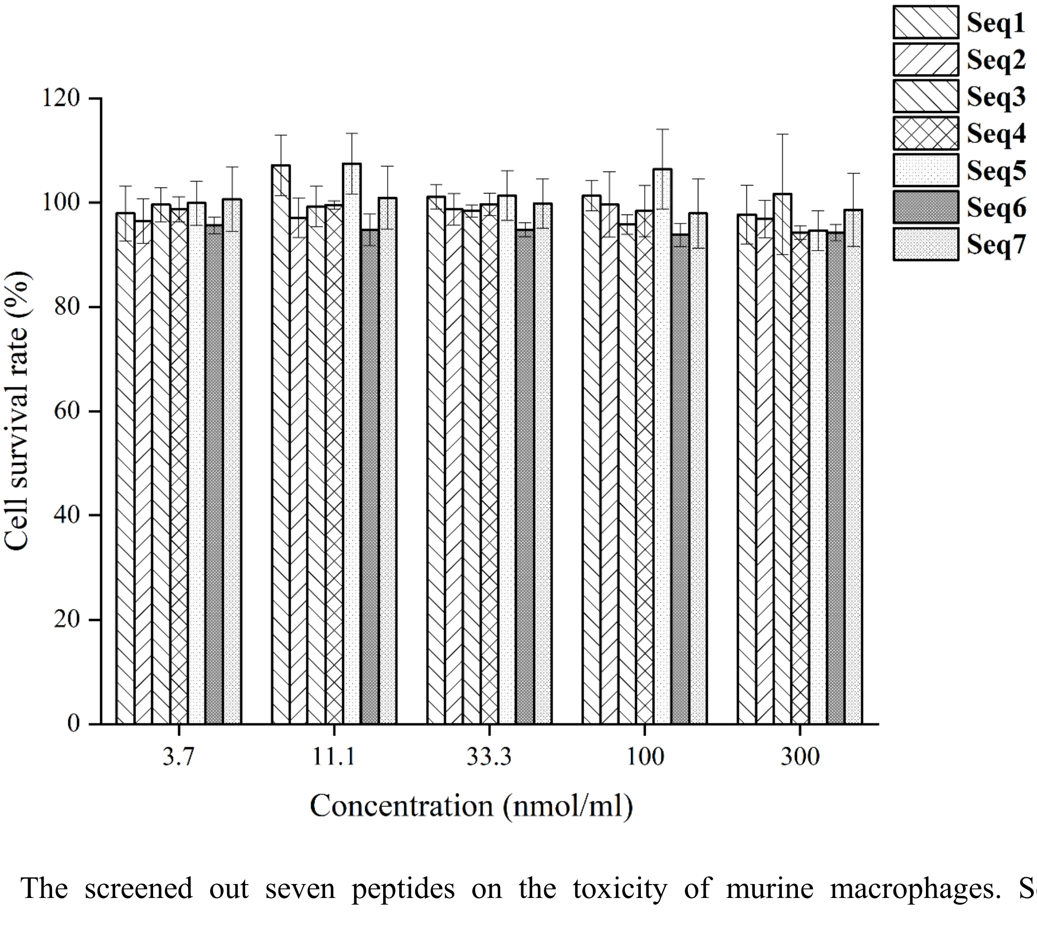

Figure S1 The screened out seven peptides on the toxicity of murine macrophages. Seq1, GGGPGPM; Seq2, GPPGPAGPAG; Seq3, FGFDGDF; Seq4, GPAGPAGPIGPVG; Seq5, AGPAGPAGPAGPR; Seq6, PGPMGPSGPR; Seq7, PAGPAGPIGPV.

2. The number of prolines in the peptide of start digestion samples (STA)

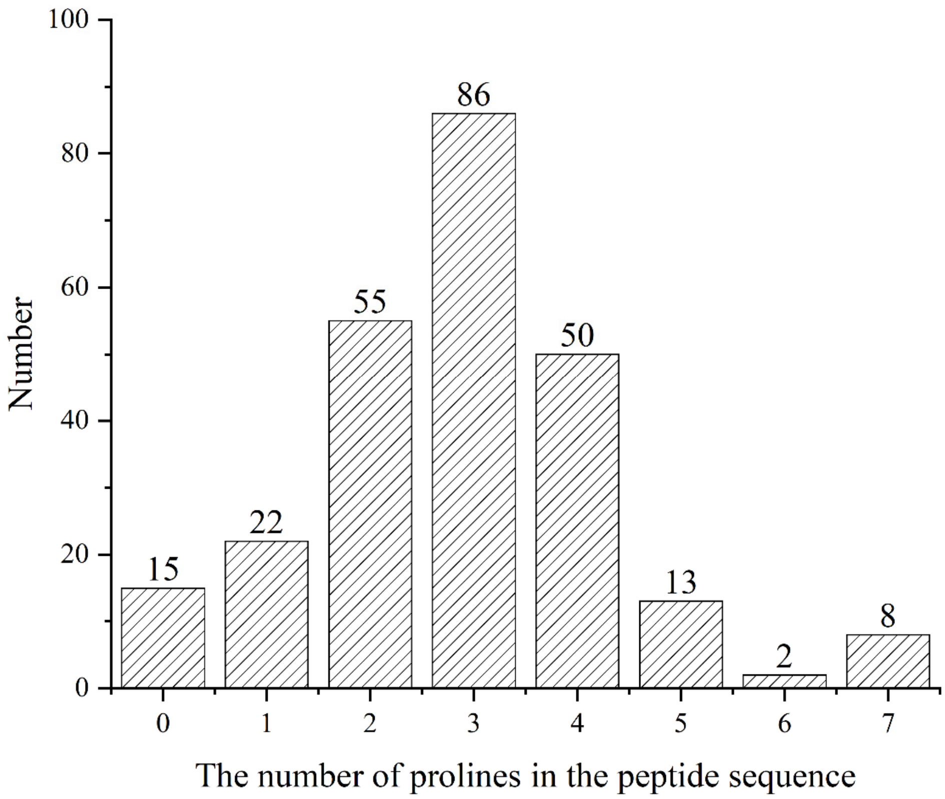

Figure S2 The number of prolines in the peptide sequence.

3. The terminal residue of peptides in start digestion samples (STA)

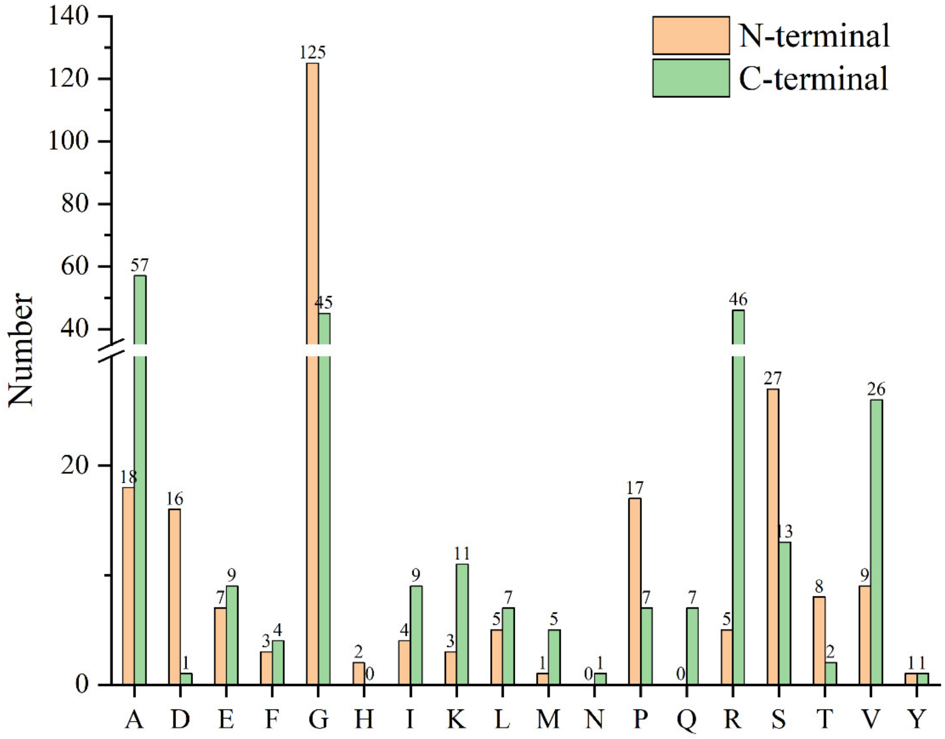

Figure S3 The terminal residue of peptides in start digestion samples

4.

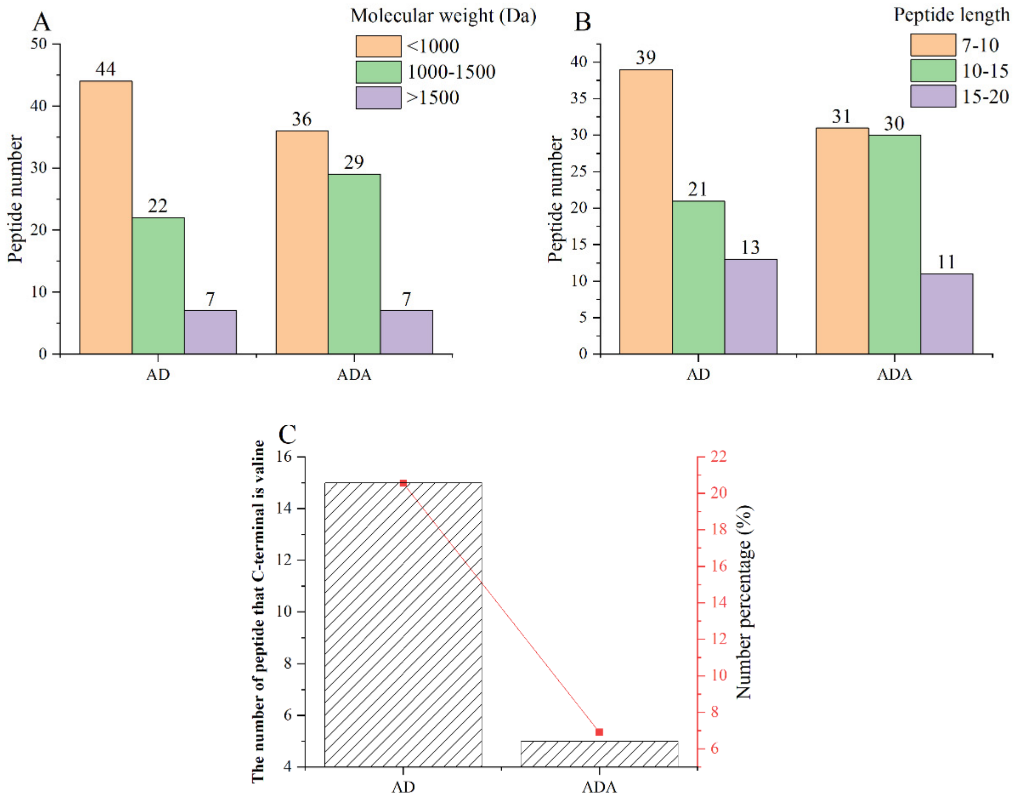

Figure S4 The statistical analysis of molecular weight distribution (A), peptide length (B), and amino acids composition (C) of peptides in the AD group and ADA group.

References:

Gao, S., Hong, H., Zhang, C., Wang, K., Zhang, B., Han, Q.-a., Liu, H., & Luo, Y. (2019). Immunomodulatory effects of collagen hydrolysates from yak (Bos grunniens) bone on cyclophosphamide-induced immunosuppression in BALB/c mice. *Journal of Functional Foods, 60*.

Ma, Y., Hou, Y., Han, B., Xie, K., Zhang, L., & Zhou, P. (2021). Peptidome comparison following gastrointestinal digesta of bovine versus caprine milk serum. *J Dairy Sci, 104* (1), 47-60.

Puchalska, P., Concepcion Garcia, M., & Luisa Marina, M. (2014). Identification of native angiotensin-I converting enzyme inhibitory peptides in commercial soybean based infant formulas using HPLC-Q-ToF-MS. *Food Chem, 157*, 62-69.

Venkatesan, K., & Nazeer, R. A. (2013). Antioxidant Activity of Purified Protein Hydrolysates from Northern Whiting Fish (Sillago sihama) Muscle. *International Journal of Peptide Research and Therapeutics, 20* (2), 209-219.

Zhao, Y. Q., Zhang, L., Tao, J., Chi, C. F., & Wang, B. (2019). Eight antihypertensive peptides from the protein hydrolysate of Antarctic krill (Euphausia superba): Isolation, identification, and activity evaluation on human umbilical vein endothelial cells (HUVECs). *Food Res Int, 121*, 197-204.
